# Of dogs and hookworms: man’s best friend and his parasites as a model for translational biomedical research

**DOI:** 10.1186/s13071-018-2621-2

**Published:** 2018-01-25

**Authors:** Catherine Shepherd, Phurpa Wangchuk, Alex Loukas

**Affiliations:** Centre for Biodiscovery and Molecular Development of Therapeutics, Australian Institute of Tropical Health and Medicine, James Cook University, Cairns, Australia

**Keywords:** *Ancylostoma caninum*, Human hookworm, Hookworm model, Translational model, Omics

## Abstract

We present evidence that the dog hookworm (*Ancylostoma caninum*) is underutilised in the study of host-parasite interactions, particularly as a proxy for the human-hookworm relationship. The inability to passage hookworms through all life stages in vitro means that adult stage hookworms have to be harvested from the gut of their definitive hosts for *ex vivo* research. This makes study of the human-hookworm interface difficult for technical and ethical reasons. The historical association of humans, dogs and hookworms presents a unique triad of positive evolutionary pressure to drive the *A. caninum*-canine interaction to reflect that of the human-hookworm relationship. Here we discuss *A. caninum* as a proxy for human hookworm infection and situate this hookworm model within the current research agenda, including the various ‘omics’ applications and the search for next generation biologics to treat a plethora of human diseases. Historically, the dog hookworm has been well described on a physiological and biochemical level, with an increasing understanding of its role as a human zoonosis. With its similarity to human hookworm, the recent publications of hookworm genomes and other omics databases, as well as the ready availability of these parasites for *ex vivo* culture, the dog hookworm presents itself as a valuable tool for discovery and translational research.

## Background

“His name is not Wild Dog any more, but the First Friend, because he will be our friend for always and always and always.” Rudyard Kipling

Over one-third of people on the planet harbour a parasitic helminth [1, 2]. Helminth infections are responsible for a host of morbidities that trap people in a cycle of poverty. Moreover, parasitic diseases kill millions of people each year, primarily in developing countries of the tropics [3, 4]. Over 80 percent of helminth infections are caused by soil transmitted helminths (STH) [5]. Elimination of STH in Western society started in the early 20th century as a result of better sanitation and public sewerage programs [6]. The problem of helminth infections in developing nations was recognised, but it was not until the advent of anthelmintic drugs and the World Health Organisation initiatives of the 1950s that widespread elimination of STH was attempted [7–10].

As we approached the 21st century, rather than celebrating the worldwide demise of STH, drug failures and incomplete coverage has meant the persistence of this foe. Previously, effective drugs like mebendazole and other benzimidazole anthelmintics are now unable to completely eliminate STH infections [1, 11–18]. Historical evidence suggests that drugs alone will not result in the elimination of STH infection and the development of resistance to benzimidazoles in gastrointestinal nematodes of livestock [19–21] is a major point of concern for human STH infections, due to the reliance on albendazole for human hookworm treatment. Indications are that other means of control should be at the fore of research and development agendas [3, 22–24]. Two such avenues are the identification of new anthelmintic compounds [25, 26] and the development of vaccines that are effective against STH [3, 27]. Key to this research is a fundamental understanding of the interactions between helminths and their environments as well as the key signals that helminths require from their hosts to facilitate their extraordinary parasitic existence [10]. Characterising parasitic helminths at a deep molecular level will reveal vulnerable pathways that can be exploited by novel drugs [10, 28], and molecules of immutable vaccine antigen potential [29, 30].

Developing nations are not the only benefactors of hookworm research. Indeed, in developed countries there is an increase in the incidence of autoimmune and allergic diseases that has been linked to lack of exposure to pathogens such as STH [31]. It is apparent from epidemiological studies [32–36] and clinical trials [37, 38] that the immunomodulatory inputs provided by exposure to STH and other pathogens assist in the development and maintenance of a healthy immune system in humans. Characterising helminth-driven immunoregulation at a molecular level will shed light on the aetiology of inflammatory diseases, and may uncover important disease/pathology pathways that can be targeted by helminth-derived therapeutics [39, 40].

The identification of a suitable model for the study of such a broad research agenda examining both the benefits of iatrogenic helminth infections and the pathogenesis of STH infections needs to be carefully considered. The benefit is the generation of a large amount of translatable research data coupled with an economy of scientific effort and resources. Fruitful models ideally need to be naturally occurring (i.e. not a human parasite manipulated to survive in experimental animals) and closely resemble the relationship between a STH and its human host. The human-hookworm-dog axis reflects an intimate evolutionary relationship between three organisms, a triad that presents a naturally selected model for integrated research. The dog hookworm, primarily *Ancylostoma caninum*, is discussed in this context in this review.

### Of humans, hookworms and hounds: the relationship between humans, hookworms and dogs is enduring and nuanced

**Fig. 1:**
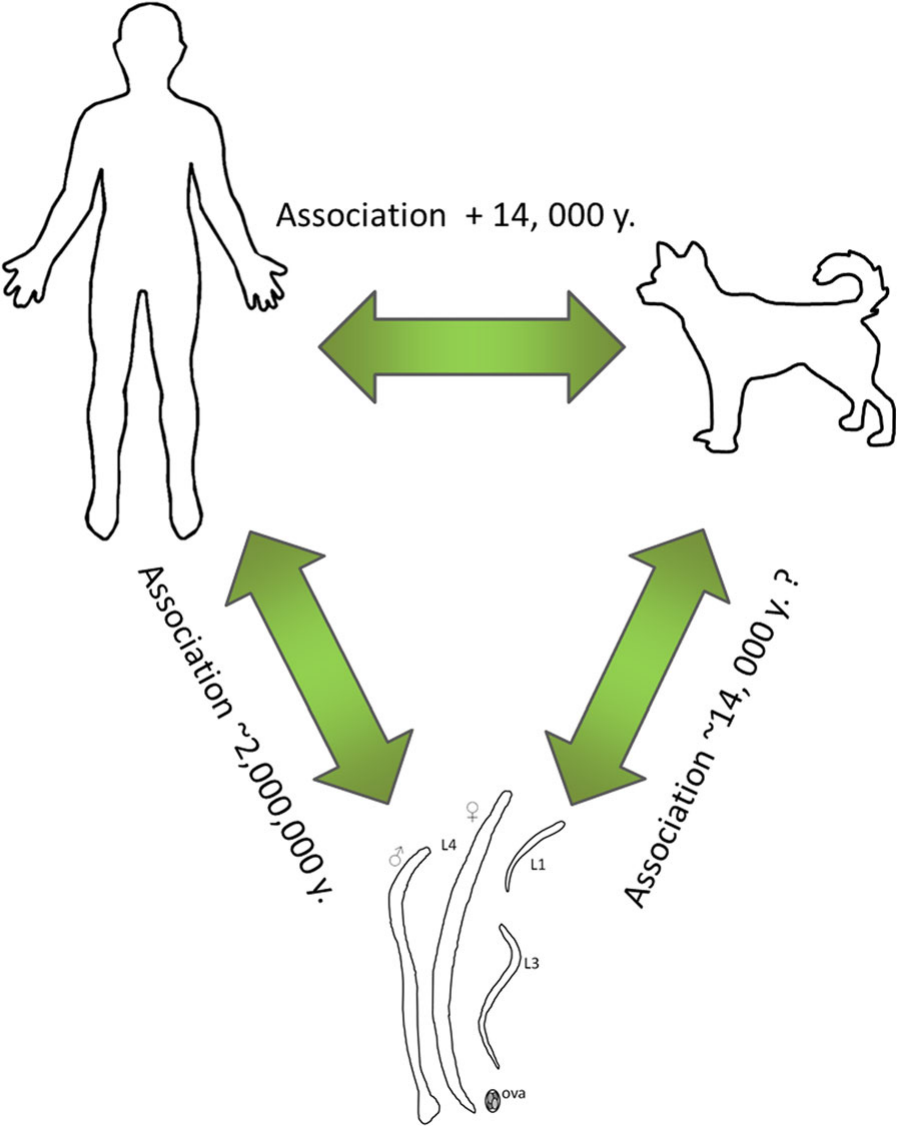
The human-hookworm-dog relationship is defined by a long association. Positive evolutionary pressure on canines has caused them to adapt to human environments. Hookworms have likely had conflicting immunogenic environments during cross infection events between human and dogs. In the ~14,000 years of association (conservative estimate) it is theoretically possible to have nearly 73,000 generations of hookworm in this timeframe

For thousands of years (estimated to be anywhere between 14,000 and ~35,000 years [41]) humans and canines have shared the same evolutionary drivers. The process of domestication has resulted in positive selective pressure on dogs to be more like humans for thousands of generations [42–44]. Genetic evidence suggests that dogs were domesticated from wolves [45], with successive domestication events occurring over at least 33,000 years [41]. Importantly, dogs accompanied humans over the threshold from hunter gatherers to agricultural based societies, and this close association with humans has meant that dogs have evolved to tolerate diets rich in starch [46]. Co-habitation between humans and dogs has resulted in shared nutritional, bacterial and pathogenic environments [42, 44, 47], making dogs a natural animal model for human disease [48]. Figure 1 illustrates the triad relationship of hookworms, humans and canines. Sharing environments with each other increases the risk of cross-infection with each other’s parasites. We know that *Strongyloides*, *Toxocara*, tapeworms and hookworms can be passed from dogs to humans and bidirectional transfer occurs in some cases, acting as pools for reinfection.

### Dogs as functional models for human disease

The parallel evolution between dog and human may have driven the predisposition to autoimmune diseases through antagonistic pleiotropy [41]. Diseases that have underlying similarities to human disease can be utilised for the study of aetiology and treatment. Genetic bottlenecks that have occurred in dog breed conformation have meant that many dog breeds are good models for both monogenic and complex genetic human diseases [48]. For example:

- Nova Scotia duck tolling retrievers develop canine systemic lupus erythematosus which is homologous to human systemic lupus erythematous (SLE) [48]
- Osteogenesis imperfecta (brittle bone disease) in beagles and golden retrievers (polygenic disease) [49, 50]
- A number of dog breeds (Pembroke Welsh corgi, Boxer, Rhodesian ridgeback, German Shepherd dog, and Chesapeake Bay retriever) develop canine degenerative myelopathy, an amyotrophic lateral sclerosis-like disease that has similar underlying genetic basis in humans (sod1 gene) [51]
- Diseases such as narcolepsy [52], cancers [53, 54] and obsessive-compulsive disorder [55] that occur in humans have the same underlying genetic basis in dogs

Diseases like diabetes that occur in both humans and dogs and are polygenic with similar underlying single nucleotide polymorphisms (SNPs) in T helper 2 type cytokines, reflect the complex environmental and genetic sequelae in the development of this disease [56]. Similarly, dilated cardiomyopathy (DCM) is associated with a large number of homologous genes in both humans and canine breeds [57], making these dogs excellent models for the study of this disease in humans. These parallels between human and dog diseases mean that canine models provide identification, diagnostic and therapeutic research opportunities in a naturally occurring animal model.

The case of dogs and inflammatory bowel disease (IBD) is a little more confounding. Dogs with spontaneous IBD have similar symptoms and clinical presentation to humans with the disease [58, 59], yet the underlying basis of these diseases has not been identified. In the case of risk for gluten sensitivity in dogs there has been no link to the major histocompatibility complex (MHC) genes [60], as has been found in humans with celiac disease [61, 62].

Despite the established value of dog-human disease models, canine models are yet to be fully exploited as functional models for human hookworm disease. One exception is the Human Hookworm Vaccine Initiative, which used the *A. caninum*-dog model [63] for identifying molecules that could be candidates for human hookworm vaccines [3].

### Hookworms are a large group of closely related organisms

**Fig. 2:**
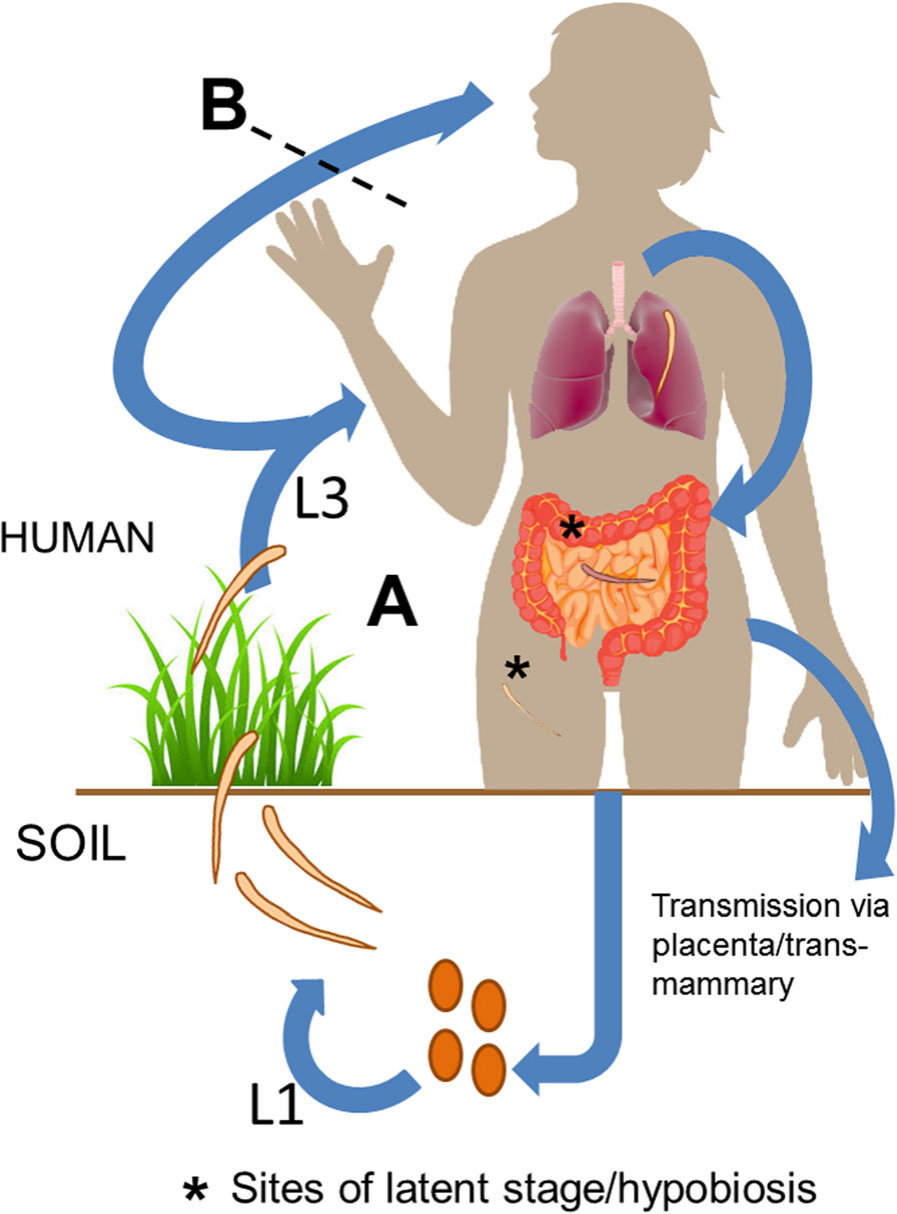
Hookworm life-cycles. Life-cycle **a** Ova contained in infected faeces hatch in soil and larvae live freely for up to two moults. Third-stage larvae (L3) come in contact with skin and penetrate the epidermis. Migrating through the lymphatic and circulatory system they end up in the lung. Larvae mature *en route* to the upper gastrointestinal tract *via* the pharynx and become fifth-stage larvae in the duodenum. Once attached to the small intestine they feed, become mature to reproductive capacity and mate. Eggs produced by the female worm are then shed in faeces. Alternatively, the free-living larvae are capable of infection through the oral route. These organisms are capable in some cases of latent stage/hypobiosis and trans-mammary or placental transmission. Life-cycle **b** does not include oral transmission. Latent stage/hypobiosis and alternative transfection routes are not reported in these organisms

In humans, the term “hookworm” is often used to refer to *Necator* and *Ancylostoma* interchangeably even though there are a number of distinctions as highlighted in their life-cycles, as described in Fig. 2. These organisms are grouped together on the basis of clinical presentation, the similarity of the organisms (both in appearance and life-cycle) and their success as parasites (Table 1). Physically, hookworms can appear similar, with *Necator americanus* and *Ancylostoma duodenale* ova being difficult to distinguish visually. Phenotypic similarities can mean that species that have large genetic separation can appear physically alike, for example *Ancylostoma braziliense* and *Ancylostoma ceylanicum* have identical mouthparts in the adult stage, making it impossible to accurately identify species without genetic tools [49, 50]. This means previous attributions of pathogenesis and distribution limits may be more complex than assumed, and in some cases flawed [50].

**Table 1:** The members of the Ancylostomatidae family and their hosts have overlapping distributions. Zoonotic disease potential, distribution and pathogenic significance are described in this table. Life-cycles A and B are described above

Species	Definitive host	Human zoonosis	Paratenic host	Global incidence	Notes	Ref.
*Ancylostoma duodenale*	Human	–	Cat, dog	Europe, Africa, India, China, Asia, Americas	Life-cycle A as described in [59]; hypobiosis; Wakana disease in humans	[159–161]
*Ancylostoma caninum*	Dog	Cutaneous infection, usually asymptomatic and arrests in tissues; adult worm in gut can cause eosinophilic enteritis	–	Tropical and subtropical regions	Life-cycle A; hypobiosis	[86, 162, 163]
*Ancylostoma ceylanicum*	Dog, cat, human	–	Rodent	Asia, India, Sri Lanka, Philippines, Australia	Life-cycle A	[151, 164–166]
*Ancylostoma braziliense*	Dog, cat	Cutaneous *larva migrans*	Rodent	Brazil, Africa, India, Sri Lanka, Indonesia, Philippines	Life-cycle A	[81, 167]
*Ancylostoma pluridentatum*	Wild cats, jaguar, leopard	None noted	–	South and Central Americas; thought to be introduced to Florida bobcat populations mid-1950s	Life-cycle A	[168, 169]
*Ancylostoma tubaeforme*	Cat, lynx	Cutaneous *larva migrans*	Rodent	Worldwide	Life-cycle A	[170, 171]
*Ancylostoma kusimaense*	Badger	None noted	Dog	Japan	Life-cycle A	[172]
*Ancylostoma protelesis*	Aardwolf	Unknown	–	Africa	nd	[173]
*Ancylostoma somaliense*	Jackal	Unknown	–	Africa	nd	[173]
*Ancylostoma genettae*	Genet (small African carnivore)	Unknown	–	Africa	nd	[173]
*Necator americanus*	Human, gorilla	Ground itch	–	Africa, India, Asia, China, Central America	Life-cycle B	[174–176]
*Necator gorillae*	Gorilla	Unknown	–	Africa	nd	[176]
*Uncinaria stenocephala*	Foxes, wolves, coyotes	Cutaneous *larva migrans*	Dog, cat, rodent	Temperate climates	Life-cycle B	[96, 177]

*Abbreviation*: *nd* life-cycle is not described in the literature

The success of hookworms as parasites hinges on their ability to be well tolerated and adaptive to their hosts. Hookworms as gut dwelling parasites gain their nutrition from their hosts in the form of blood [62, 63]. To do this they attach to and slice through the gut wall where they feed. Ancylostoma is a voracious feeder, estimated to consume between 0.15–0.23 ml blood/worm/day, whereas Necator consumes ~0.03 ml blood/worm/day [64]. The host reacting to injury and the presence of the parasite attempts to expel the parasite through innate immune responses such as mucus production, increased peristalsis and eosinophilia [65–67]. Despite this, hookworms are usually well tolerated in a healthy host [68, 69] because they actively create an area of immune privilege around the site of their attachment by secreting a suite of immunomodulatory molecules [39, 70–72].

The hookworm genera *Ancylostoma* and *Necator* are intimately associated with the history of humanity, and it seems these heirloom parasites have evolved with humans from our primate ancestors [51, 52]. Such is the close relationship that hookworm ova have been used in archaeology to help identify migration routes of humans [51–53] and supports the intertwined immunomodulatory interactions proposed by Rook’s “Old Friends Theory” [31]. In a literature survey of *Ancylostoma* and related species (Table 1), we identified a number of taxonomically related hookworms. These hookworms occur across a wide range of hosts with some species capable of establishing patent infection in dogs, cats, golden hamsters and humans (as is the case for *A. ceylanicum*). The common dog hookworm (*A. caninum*) is closely related to the human hookworm species *N. americanus* and *A. duodenale*. Phylogenetic studies highlight the close relationship between *A. caninum*, *N. americanus* and *A. duodenale* [54–58]. In addition, the life-cycles of *A. caninum* and *A. duodenale* are very similar [59]: both have a latent stage, faecal-oral transmission, larval hypobiosis and mammary-oral infection. The presence of a latent stage is important as these organisms have a predisposition to chemotherapeutic resistance [60] and are useful for the screening of anthelmintic drugs [61].

### The dog hookworm has well-described physiology and biochemistry which situates it as a translational model

The dog hookworm, *A. caninum* was described in 1859 by Ercolini, and its physiology, morphology, biology and epidemiology have been well described, making it a good candidate as a model for translational research. Initial studies focussed on the description, harvesting and optimisation of culture conditions for physiology experiments. It was determined that optimal conditions for harvested hookworms was a Krebs-Ringer solution with the addition of 50% dog serum, allowing adult worms to survive for up to 10 weeks [62]. These hookworms were able to reproduce and produce viable eggs. The addition of whole red blood cells did not have any effect on feeding activity and survival [62], and it was shown by other investigators that the majority of red blood cells pass through hookworms intact [63]. It was from these observations that it was assumed that hookworms get the majority of their nutrition from plasma rather than erythrocytes.

It is recognised that parasites convert their metabolic processes as they transition from free living to parasitic life-styles. However, little is known about the host cues and parasite molecular pathways which govern this process. Metabolic labelling studies in dog hookworm have determined that C^14^-labelled glucose is not converted to glycogen [63], but is diverted into amino acid production [64] in a metabolism dominated by fermentative processes [65, 66]. Exposure of hookworm L3 to dog sera increases feeding rates [67], and large diffusible solutes (protein fraction) stimulate the consumption of glucose [62]. Cyanide inhibition studies show that the adult dog hookworm is capable of aerobic metabolism and has a tricarboxylic acid (TCA) cycle that is able to oxidize both pyruvic and succinic acids [68]. In addition, NADH respiration is not strongly coupled to oxidative phosphorylation, with evidence that hookworms lack respiratory control [69]. Adult *A. caninum* succinoxidase activity does not seem to be tightly coupled to the synthesis of ATP, and external NADH oxidation that is not coupled to phosphorylation can occur. The low ratios of phosphorylation/oxidation may reflect loosely coupled pathways of respiration or the existence of two pathways of respiration, one coupled to the esterification of inorganic phosphate and another to the NADH pathway [69].

Fatty acids produced by *A. caninum* are major end products of metabolism. Labelling studies have shown that glucose is metabolised to produce acetate, propionate and CO_2_ while the amino acids L-valine and L-leucine are the precursors of isobutyric acid and isovaleric acid, respectively [65]. *Ancylostoma caninum* adult worms consume larger quantities of oxygen than other intestinal nematodes [67]; however, they have been shown to fix CO_2_ to produce propionate from amino acids *via* a CO_2_ fixation pathway [65, 67]. Adult hookworms display a slowing or cessation of metabolic fermentation in the presence of oxygen [65], and the presence of oxygen inhibits anaerobic glycolysis. The interactions of the end products of hookworm metabolism with the host have never been explored. Similarly, there has been no investigation of the role of secondary metabolites produced by hookworms and their role in the host-parasite relationship.

Studies of hookworm transition through developmental stages have failed to comprehensively identify the triggers that control the penetration, migration and maturation in host tissues. Like the human hookworm *A. duodenale* [70], *A. caninum* in dog somatic tissues can enter hypobiosis, a hibernation-like state often compared to dauer formation in the free-living nematode *Caenorhabditis elegans* [71, 72]. Experimental infection of dogs with hookworms has increased our understanding of these processes. For example, experimental infection of dogs with *A. caninum* results in a greater proportion of parasites proceeding to hypobiosis in dogs which are immunosuppressed by corticosteroid treatment, and cessation of treatment results in resumption of parasite development, albeit in a relatively unpredictable manner [61]. The cause of this resumption is unknown but it implies complex molecular/physiological interactions between host and parasite through all stages of development.

The propensity to hypobiosis is a valuable characteristic of a model because hypobiotic larvae are less susceptible to anthelmintic drugs [60], presumably due to their ability to slow their metabolic processes. In vitro studies of *A. caninum* show recovery from hypobiosis can be induced by exposure to muscarinic acetylcholine receptor agonists and cyclic GMP. This activation process results in a rapid release of infection-associated proteins [71, 73, 74], which in part mimics the host-specific signalling events seen in L3 upon exposure to host serum [74]. Very little is known about the L3 lung stage of *A. caninum* or the triggers that prompt migration and maturation of the L4 as it arrives in the gastrointestinal tract and reaches sexual maturity in the small intestine. Recent reports show that *A. caninum* in older dogs induces a tolerogenic area around the site of attachment in the gut [75]. In addition, we know from proteomic and more targeted studies that *A. caninum* produces a number of proteins that putatively interact with immune cells and can induce anti-inflammatory responses [76–80] which in turn promotes tolerance in the host.

Our understanding of *A. caninum* as a human zoonosis has also changed over time. Initially the zoonosis was defined by *larva migrans* where the parasite could penetrate the epidermis but fails to successfully migrate and is trapped in the skin and underlying musculature, leading to localised irritation and pruritus [81–83]. It has now been shown that *A. caninum* is able to complete its migration in humans, with occasional worms reaching the human gut, although a patent human infection with *A. caninum* has yet to be reported. Studies in mice, a monkey and a cat have shown migration of the parasite into muscle tissues with little inflammatory response [84]. This phenomenon has also been reported in humans with migration of hookworms to muscle tissues in an individual with a large cutaneous exposure [85]. In 1994, Croese [86] described numerous cases where solitary *A. caninum* were identified in the human gut by endoscopy, discovered during clinical investigation for eosinophilic gastroenteritis [87]. These observations indicate that *A. caninum* could be a suitable proxy for human-hookworm interactions, and much can be learned about human hookworm infection by studying *A. caninum* in its natural dog host.

### Omics can provide useful information but must be augmented with information from animal models

Omics is the collective term for the characterisation of an organism at the genome, transcriptome, proteome and metabolome levels. Increasingly, omics have been used to guide research in host-parasite biology and therapeutics discovery. This approach has been used in the case of parasitic trematodes to identify the mechanisms of drug resistance and to identify novel drug targets [88]. Metagenomics and metabolomics are the newest and most rapidly advancing ‘omics’ technologies, and offer under-utilised promise for understanding the molecular basis of host-helminth interactions, particularly metabolism, pharmacotherapeutic action [89] and the presence and function of commensal microbes. The limitation of comparative omics is that often there are no homologs or orthologs of helminth molecules (particularly proteins) in other organisms to assign putative function, and many of the metabolites are of unknown origin and function. For example, there are over 700 transcripts in the *A. caninum* transcriptome encoding for proteins with secretory signal peptides with no function assigned [90]. Unannotated genes in parasites are of interest due to their likely role in governing a parasitic existence [91]. To augment omics databases to allow for meaningful bioinformatics analyses, animal models provide invaluable information. These models provide important bioactivity information that cannot be inferred by bioinformatics alone [39].

We have assembled the currently available omics information for the family Ancylostomatidae in Table 2, and have included for comparison the free-living model nematode *C. elegans* and the filarial nematode *Brugia malayi*. By using comparative tools it was possible using these databases to identify genes putatively associated with parasitism by comparing the different transcriptome patterns of the three helminth species [90]. Mining of assembled proteomic databases can help to identify proteins, but often the target protein is of unknown. For example, using a shotgun proteomics approach Mulvenna et al. [76] identified 105 *A. caninum* ES proteins increased recently to 315 putative proteins in our laboratory [92]. Based on gene ontology alone many of these are putative or of unknown function [76, 92].

**Table 2:** The current omics status for the Ancylostomatidae, the free-living model nematode *Caenorhabditis elegans*, and the filarial nematode *Brugia malayi*. Genome and transcriptome progress for Ancylostomatidae can be monitored on the Gold-genome online database (https://gold.jgi.doe.gov/distributiondata?domain=EUKARYAL&rank=family&group=Ancylostomatidae.). Transcriptome, proteome and metabolome database updates can also be monitored on (http://www.genome.jp/kegg/kegg1.html) and Wormbase; (www.wormbase.org)

Organism	Genome	Transcriptome	Proteome/secretome	Metabolome	Reference
*C. elegans*	×	×	×	×	[178–187]
*N. americanus*	×	×	×	−	[93, 130, 132, 188–192]
*A. duodenale*	−	−	−	−	−
*A. caninum*	×^a^	×	×	−	[76, 90, 92, 188, 193–195]
*A. ceylanicum*	×	×	−	−	[193, 196]
*A. braziliense*	−	×	−	-	[197]
*Brugia malayi*	×	×	×	−	[9, 90, 198–203]

^a^Data collection still in process

*Key*: ×, omics data available; −, omics data not available

Current databases for helminth omics, particularly hookworm, are incomplete. Only *N. americanus* has a published genome [93], and only *A. caninum* has a comprehensive secreted proteome from the adult worm [76], although the somatic adult worm proteome has been reported from *N. americanus* [93]. The plasticity and adaptability of hookworms further accentuates the deficit in bioinformatics data. Significant subpopulation variation of *A. caninum* has been observed [94], and it has been speculated that this variation may indicate speciation events. In addition, these subpopulations could reflect differences in infectivity for *A. caninum* [95]. The analysis of restriction fragment length polymorphisms (RFLP) of helminths from India and Vietnam supports the observed phylogenetic relationships but also identifies unclassified *Ancylostoma* species [96, 97]. This pattern is also observed with *N. americanus* [98, 99]. The source of hookworm material used to generate different omics databases is usually restricted to small populations, often passaged through unnatural hosts, and different populations are frequently used to compile different datasets (see Table 2). In conjunction with the known plasticity of hookworm species, these factors may diminish valid comparisons across databases. By using *in silico* techniques it is possible to use comparative bioinformatics to combine proteomic and transcriptomic databases to determine metabolic pathways [100].

Combining omics data with functional data from animal models is a very powerful tool to identify critical physiologic and metabolic pathways. Helminth genomes are often poorly annotated, which can result in missed identifications of novel pathways in parasites. For example, it is only from metabolic labelling of metabolic intermediates of the *Leishmania* parasite promastigotes that it was possible to show that the parasite pooled reservoirs of essential molecules for the TCA cycle through the fermentation of succinate [28]. This is a novel pathway that could potentially be used as a target for anti-parasitic drugs and provides an understanding of how parasites cope with changes in their environment. The metabolome of *Entamoeba* during encystation shows the presence of polyamines [101], an unexpected finding given that the enzymes responsible for polyamine biosynthesis are not annotated in the genome [102]. Eicosanoids in helminths are likely produced by novel pathways, as bioinformatics searches for the genes encoding homologues of the mammalian enzymes in this biosynthesis pathway have not been identified [103]. Given these examples, and the adaptability of the hookworm and its phenotypic plasticity, metabolomics profiling is likely necessary to uncover novel pathways and molecules used by these parasites [104].

### Animal models of hookworm interaction

One of the major issues with the study of hookworms is the inability to culture these parasites through all developmental stages of their life-cycle in vitro. To this end, animal models are employed. The ideal model for human hookworm infection should reflect the pathophysiology of the helminth-host interaction and be experimentally accessible. Current models all have limitations and many animal experiments are considered "under-powered" [105] due to the ethical restrictions on animal experimentation numbers. A further restriction on models is the availability of immunological reagents for particular species. Reagents are readily available for humans, mice/rats, and to a lesser extent dogs and hamsters, but are more restricted for cats, which only have a limited palette of immune reagents. Moreover, human hookworms do not generally reach maturity in mice or rats [106, 107], precluding functional studies using genetically modified hosts.

There are three main experimental animal models commonly used as a proxy for gastrointestinal (GI) nematode infections of humans; mice/rat, hamsters and dogs as summarised in Table 3. There have been attempts to establish hookworm-primate models using marmots [108] however these models have been constricted by experimental cost [109]. The two benchmark murine models used for STH infection discussed in this review are *Heligmosomoides polygyrus bakeri* and *Nippostrongylus brasiliensis*. *Trichuris muris* is homologous to the human whipworm *Trichuris trichiura*, and while it is a STH it is not within the scope of this review. The rat is the natural host of *N. brasiliensis* and is considered a good model for human hookworm infection, particularly due to the similarities between the *N. brasiliensis* and *N. americanus* secretomes [110]. However, there are some features of the *N. brasiliensis* life-cycle in rodents that are dissimilar to human hookworm infections, particularly the rapid transit time from percutaneous infection to reaching the gut, as noted below. *Nippostrongylus brasiliensis* is not a natural parasite of mice, although it can complete its life-cycle in them.

**Table 3:** The major animal models used to study human hookworm infection

Animal model	Naturally occurring host-parasite	Similar life-cycle cf. *Necator/Ancylostoma* spp. in humans	Infection model	Model used to study	Regulatory restrictions	Reference
Mouse (*Mus musculus*)						
* Heligmosomoides polygyrus bakeri*	Yes	No (no lung stage)	Chronic helminthiasis	Pathophysiology of infection, immunological studies, vaccinomics	–	[114, 115, 204–208]
* Nippostrongylus brasiliensis*	No	Yes	Rapid expulsion	Immunology of helminth infection, pathophysiology of infection	–	[110, 207, 209–212]
Golden Syrian hamster (*M. auratus*)						
*Ancylostoma ceylanicum*	No	No (infection by oral gavage)	Rapid expulsion	Vaccinomics, anti-helminthics	Hamsters not permitted in some countries (e.g. Australia)	[123, 213, 214]
*Necator americanus*	No	Yes	Chronic helminthiasis	Pathophysiology of infection, immunological studies, vaccinomics	Hamsters not permitted in some countries. Ova transport restricted in US	[121, 215, 216]
Beagle^a^ (*Canis lupus familiaris*)						
*Ancylostoma caninum*	Yes	Yes including hypobiosis	Acute and chronic helminthiasis	Pathophysiology of infection, immunological studies, vaccinomics	Ethical considerations	[144]

^a^Alternative sources of *A. caninum* can be from naturally infected dogs in endemic areas

While *N. brasiliensis* is less closely related phylogenetically to human hookworms than are other *Ancylostoma* spp., it is often used to study the pathophysiology and immunology of STH infection. One of the major differences between human hookworm infection and *N. brasiliensis* infection in mice is the self-cure phenomenon that occurs. Within two weeks of infection, mice expel the parasite and develop protective immunity that is dependent on CD4^+^ Th2 cells. Indeed, this model has been used to study the development of host protective immunity and the in vivo regulation of Th2 responses in general [111–113]. One of the advantages of this model is the minimal inter-animal variation, however, its life-cycle is abbreviated and is not characterised by the chronicity of human hookworm infection.

As a natural parasite of mice, *H. polygyrus* is often chosen to study the pathophysiology and the immunology of intestinal nematode infections [111, 114, 115]. While taxonomically related to human hookworms, *H. polygyrus* differs from human hookworms in that it has a much simpler life-cycle. *Heligmosomoides polygyrus* does not have a lung stage and exhibits an enteric life-cycle with a faecal-oral infection route. However, like human hookworm infection, *H. polygyrus* establishes a chronic infection in its definitive host. As such, this model has been used to extensively characterise the Th2-skewing and immunoregulatory properties of GI nematodes [116], and ultimately resulted in the introduction of the term “modified Th2 response” [117].

The mouse models of *N. brasiliensis* and *H. polygyrus* have been used to decipher the innate and adaptive immune responses responsible for worm expulsion and the development of chronicity [118]. Both parasites drive a modified Th2 response typified by IL-4, IL-5, IL-9, IL-13 and IL-10 which mediates eosinophilia, mucosal changes such as mast cell hyperplasia, mucus production and IgG1 and IgE production [119].

One of the most important breakthroughs in the development of animal models for human hookworm infection was the establishment of patent infections in golden Syrian hamsters (*Mesocricetus auratus*) with *N. americanus* and *A. ceylanicum*. Both models however are “labours of love” and require considerable time to establish because they generally rely on immunosuppression with steroids, at least in the early phases of model establishment [120–122]. Immunosuppression with steroids of the golden Syrian hamster allows *A. ceylanicum* and *N. americanus* infections to reach patency [121, 123], thereby providing material for laboratory analyses including genomic, transcriptomic and metabolomics studies. The *A. ceylanicum-*hamster model [124] has been utilized for testing vaccine efficacy, understanding mechanisms of adaptive immunity, and testing of anthelminthic drugs, often as a model for human infection with *N. americanus* [106, 125]. These interpretations have been supported by observations in small scale studies in humans [126–128].

The *N. americanus*-hamster model was first established by Sen & Seth in 1970 [129], and tailored as a model for testing drugs and vaccines by Jian et al. in 2003 [121]. Importantly, the in vivo generation of adult-stage *N. americanus* enabled the sequencing of the adult worm transcriptome [130], and ultimately contributed material to the sequencing of the genome in 2014 [93]. It should be noted that *N. americanus* was adapted to hamsters through 100 generations of selective pressure. The ability of a complex multicellular pathogen to adapt to a new host within 100 generations reflects a high evolutionary rate also observed in *A. caninum* and reflected by high DNA polymorphism [90]. We currently do not understand the molecular basis of the adaptation of *N. americanus* to the golden hamster*.* A comparative analysis of mitochondrial cytochrome oxidase from *N. americanus* obtained from hamsters and natural human infections suggests a severe genetic bottleneck in hamster-adapted *N. americanus* which may limit the utility of this model [131]. To date, there has been no investigation of the differences in genomes between *N. americanus* sourced from hamsters and those from the native human host. The ‘omics’ information garnered from such a comparison would be useful to highlight those genes related to host adaption and could also enhance our understanding of the metabolites produced by a system in response to changes in the host and an environmental stimulus.

There are a number of caveats to consider in the hamster model of human hookworm infection. Interpretation of data generated in the *N. americanus*-adapted hamster model may be misleading when applied to human hookworm host-parasite biology due to the undefined changes discussed above. In addition, the model using immunosuppressed hamsters and *N. americanus* is still employed [132, 133], so care should be taken when interpreting results and drawing parallels between the two models. Another problem with the hamster model of *N. americanus* infection is its restricted geographic use; for example, hamsters are a prohibited laboratory species in Australia due to strict quarantine restrictions [134]. This limits its ability to be used in comparative and translatable studies universally. Logistically, the hamster is a small animal so the parasite load that the animal can tolerate is low when compared to a human or larger animal such as a dog. Moreover, adult *N. americanus* do not reach full size in hamsters [121], even though they do become fecund and infections are patent. A further weakness of this model is that the small numbers of adult parasites that can be supported in hamsters are rapidly expelled compared to the chronicity that develops in human infection [135].

### The canine-hookworm model

The gut-dwelling, blood-feeding stage of *A. caninum* is well described at biochemical, transcriptomic and immunological levels as discussed here. The excretory/secretory (ES) component of *A. caninum* (AcES) at the host-parasite interface has been described, with over 100 proteins identified in 2009 [76], many of which were ascribed to either feeding [136–138] or putatively evading the host and inducing tolerance [75, 78]. The immunomodulatory nature of AcES has been demonstrated [77, 139] with an increasing interest in translational applications for components from AcES [80, 136, 140–142]. In addition, possible mechanisms of resistance [15] and hypobiosis [84] have been studied in this model. The availability of such an accessible and defined model is attractive for a gamut of research agendas.

A major advantage of the *A. caninum*-dog relationship as a model for human hookworm infection is that it is naturally occurring, and moreover, *A. caninum* and *A. duodenale* are very similar morphologically and genetically. Due to the larger size of the animal, dogs are more suitable for recovering larger quantities of adult hookworms for laboratory studies. From an individual dog it may be possible to recover thousands of adult worms, allowing for comprehensive studies to occur from a single collection. One of the major objections to the use of dogs as animal models however is the ethical concern around their experimental use; this, combined with the expense of keeping such a large animal for research purposes, often limits the use of the *A. caninum*-canine model. Attempts have been made to create an *A. duodenale*-dog model for research purposes. Using immunosuppression with prednisolone it was possible to achieve patency within six generations of beagle [143]. In later experiments *A. duodenale* was maintained in laboratory-raised beagles for 30 generations using decreasing amounts of prednisolone, and adaption to the dog was presumed due to observed decreases in the prepatent period for the parasite, however a fully permissive model was not achieved due to the prohibitive resource demands of the project [144]. This is a common theme with large animal models.

Puppies usually harbour the heaviest burdens of *A. caninum*, and worm numbers (or at least eggs in the faeces) tend to diminish over time. An efficacious hookworm vaccine based on irradiated parasites was developed for dogs but was discontinued soon after reaching the market due to its poor shelf life and low uptake by pet owners [145, 146]. The dog hookworm vaccine was based on radiation-attenuated L3 [146], and although the protective antigenic components were not identified, two immunodominant L3 secreted antigens were described from dogs that received the vaccine [147]. Understanding the mechanisms by which vaccine-induced immunity to the dog hookworm develops could inform efforts to develop a subunit human hookworm vaccine. Such studies have guided antigen discovery for human hookworm vaccines [148], and even underpin proposed human trials with irradiated *N. americanus* L3 (AL, unpublished).

It is our experience that the dog-hookworm model can be very fruitful when *A. caninum* is sourced from areas where dogs are naturally infected, have not received recent anthelmintic therapy, and dogs are excess to requirements. These unwanted animals can result from wild dog eradication programmes, the culling of rural/remote camp or village dogs, or the sourcing of stray or unwanted dogs from residential communities in tropical and sub-tropical environments. The benefit of this approach is three-fold: (i) ethically, these animals are not experimentally infected or euthanized for research purposes, so ethical concerns on their use are minimised; (ii) international partnerships between developed and developing countries to source these hookworms means that educational/research facilities can be meaningfully engaged in STH research; and (iii) harvesting of animals from diverse geographical areas gives power to observations as it reflects natural host-parasite interactions and natural spatio-temporal variation.

The opportunity for international partnering is emphasised in the modern research agenda as it aids capacity building in developing countries [149] that are most affected by STH. It enables the development of collaborative long-term partnerships and stewardship over research agendas. This is particularly important in the case of human hookworm research as there are direct benefits to both developed and developing nations in terms of vaccine and drug development [150], as well as development of novel helminth-derived anti-inflammatory therapeutics [80]. Additionally, partnering provides the ability to extend research capabilities in a time of increasing economic rationalisation and competitive grant processes. The inclusion of a broad base of expertise from immunology, parasitology, veterinary and agricultural sciences, public health and medicine is possible in these partnerships and is often aligned with current integrated approaches to research programs.

### *Ancylostoma ceylanicum* as a model for human hookworms

*Ancylostoma ceylanicum* is one of the most indiscriminate hookworms, with the ability to naturally infect dogs, cats, humans and other animals. It is a dominant hookworm in Asia and present throughout Australasia [151, 152], and a permissive model in hamsters is available. To date, models utilising hamsters and mice infected with *A. ceylanicum* have been used to study pathogenesis [123]. This hookworm has the potential to be valuable in comparative ‘omics’ studies. For example, using transcriptomics approaches, putative anthelmintic drug targets were identified [153]. Comparing the transcriptomes of adult *A. ceylanicum* hookworms isolated from feline, canine and other animal infections has the potential to identify molecules that are responsible for the promiscuity in host preference for this parasite. Like in *A. caninum* infection, dogs infected with *A. ceylanicum* develop functional immunity to the parasite [154]. The advantage of *A. ceylanicum* as a model parasite is that it regularly reaches patency in both humans and dogs [151, 155].

*Ancylostoma ceylanicum* is closely related to both *A. duodenale* and *A. caninum* [55, 156], so the molecular mechanisms by which they regulate and skew the host’s immune pathways are likely to be shared between species. However, observations of other parasites show there are a wide number of adaptions to the parasitic life-style [157]. For example, there are classes of parasite proteins that have been implicated in host-parasite interactions with many having pleiotropic actions [39, 157, 158]. A comparative study of *A. ceylanicum*, *A. caninum* and *A. duodenale* may reveal unique adaptions for each of these closely related species.

Phenotypically, *A. ceylanicum* and *A. brasiliensis* can be easily confused, making it necessary to augment collection of parasites from natural infections with molecular characterisation [96, 151]. This complicates acquiring hookworms from naturally infected dogs where the distribution of these hookworms overlaps. The secretome has yet to be defined at the protein level for *N. americanus*, *A. duodenale* or *A. ceylanicum*, limiting the ability to make confident assumptions about sequence conservation of secreted proteins between these species. It is for these reasons that *A. ceylanicum* is a more limited model hookworm.

## Conclusions

In the context of human hookworm research, the human-hookworm-dog axis provides one of the most unique experimental opportunities for the identification of bioactive molecules for development of novel immunotherapeutics, subunit vaccines, and selection of drug targets. Domestication has meant that the unique evolutionary relationships between dogs and humans can be exploited as natural models for human diseases, notably from our perspective, the parasitic helminth infections of humans. Positive selection pressure and breed conformation has led to genetic restriction in canines, and has meant that many diseases have the same underlying genetic basis in dogs and humans. It is likely that humans and dogs have shared parasites for millennia, making them an ideal model for the study of host-parasite biology. Laboratory evidence shows that with the administration of immunosuppressive therapy it is possible to achieve patency of hookworm infections in a variety of unnatural definitive hosts. There are a number of available models for human hookworm infection; however, all of these models have limitations. Mouse models are universally available yet suffer from a range of dissimilarities to the natural human hookworm infections. Hamster models have an advantage over mouse models in that they are permissive for the human hookworms *N. americanus* and *A. ceylanicum*, but resistance to infection develops rapidly, unlike human hookworm infections. *Ancylostoma caninum* in the dog is a robust, naturally occurring relationship, and parasites isolated from animals in endemic areas have the potential to provide powerful molecular information that is unavailable from other sources, and reflects naturally occurring interactions. The use of experimentally infected large animal models for such studies is fraught with ethical considerations and high economic costs. The study of natural interactions has the potential to provide more powerful observations than those of current laboratory models. Sourcing naturally infected animals provides an opportunity for international partnering and an opportunity for a diverse portfolio of translatable research outcomes. It negates many of the ethical and cost concerns rightly expressed over the housing of large mammals. By using a strong model for sourcing parasite material it is possible to make reliable and translatable observations about hookworm host-parasite biology as well as facilitating programs focused on development of new control strategies aimed at eliminating, or at least mitigating, hookworm infection.

## Abbreviations

AcES: *A. caninum* ES component
ATP: Adenosine triphosphate
CGMP: Cyclic guanosine monophosphate
CO_2_: Carbon dioxide
DCM: Dilated cardiomyopathy
DNA: Deoxyribonucleic acid
ES: Excretory/secretory
IBD: Inflammatory bowel disease
MCH: Major histocompatibility complex
NADH: Nicotinamide adenine dinucleotide
RFLP: Restriction fragment length polymorphisms
SLE: Systemic lupus erythematous
SNPs: Single nucleotide polymorphisms
STH: Soil-transmitted helminths
TCA: Tricarboxylic acid
